# BONE MASS BY QUANTITATIVE ULTRASOUND OF FINGER PHALANGES IN YOUNG KARATE PRACTITIONERS

**DOI:** 10.1590/1984-0462/;2017;35;4;00003

**Published:** 2017-09-21

**Authors:** Camila Justino de Oliveira Barbeta, Ezequiel Moreira Gonçalves, Keila Donassolo Santos Ribeiro, Roberto Ribeiro, Everton Paulo Roman, Gil Guerra-Júnior

**Affiliations:** a Universidade Estadual de Campinas (UNICAMP), Campinas, SP, Brasil.; b Centro de Investigação em Pediatria (CIPED), Campinas, SP, Brasil.; c Universidade Federal de Santa Catarina (UFSC), Florianópolis, SC, Brasil.

**Keywords:** Children, Adolescents, BMI, Finger phalanges, Ultrasonography, Bone density, Crianças, Adolescentes, IMC, Falanges dos dedos da mão, Ultrassonografia, Densidade óssea

## Abstract

**Objective:**

To evaluate bone mass by quantitative ultrasound of the phalanges in young karate practitioners compared to a control group.

**Methods:**

Sample composed of 162 karate practitioners (52 females) and 326 healthy controls (110 females) aged 6 to 16 years old, in Western Paraná (Southern Brazil). Weight, height, BMI, amplitude-dependent speed of sound (AD-SoS) and bone transmission time (BTT) were evaluated. BMI, AD-SoS and BTT values were converted to Z scores. Mann-Whitney, chi-square or Fisher Exact tests and multiple linear regression were applied, with significance level set at *p*≤0.05.

**Results:**

Both genders showed higher values of BTT as Z scores when compared to control group. Females from the control group had higher AD-SoS values (m/s and Z score) compared to female karate practitioners. When relative and absolute frequencies were assessed according to BTT Z score in both groups, male karate practitioners’ bone mass was shown to be adequate more frequently. In female practitioners, age and weight were independent predictors of AD-SoS (R^2^=0.42) and BTT (R^2^=0.45), respectively. Among male karate practitioners, age was related to 26% of AD-SoS variances and height was responsible for 36% of BTT variances.

**Conclusions:**

Children and adolescents who practice karate were shown to have more bone mass in comparison to the control group, regardless of gender. BTT was more sensitive for this evaluation.

## RESUMO

**Objetivo:**

Avaliar a massa óssea pela ultrassonografia quantitativa de falanges em jovens praticantes de karatê em relação a um grupo controle.

**Métodos:**

Amostra constituída por 162 praticantes de karatê (52 meninas) e 326 controles escolares (110 meninas) saudáveis, de 6 a 16 anos de idade, do oeste do Paraná. Foram avaliados peso, estatura, índice de massa corporal (IMC), *Amplitude Dependent Speed of Sound* (AD-SoS) e *Bone Time Transmission* (BTT), e os valores de IMC, AD-SoS e BTT transformados em escore Z. Aplicaram-se testes de Mann-Whitney, qui-quadrado ou Exato de Fisher e regressão linear múltipla, sendo significante *p*≤0,05.

**Resultados:**

Para ambos os sexos, os praticantes de karatê apresentaram valores superiores do escore Z do BTT comparados aos controles. Quanto à AD-SoS, as meninas do grupo de controle apresentaram valor absoluto e de escore Z superiores aos apresentados pelas praticantes de karatê do mesmo sexo. Ao avaliar a frequência relativa e absoluta de acordo com o escore Z do BTT em ambos os grupos, os meninos praticantes de karatê apresentaram maior frequência de massa óssea adequada. Nas meninas praticantes de karatê, a idade apresentou poder de explicação de 42% na variação da AD-SoS e o peso de 45% na variação do BTT. Nos meninos praticantes de karatê, a idade apresentou poder de explicação de 26% na variação da AD-SoS e a estatura 36% na variação do BTT.

**Conclusões:**

Nesse grupo de crianças e adolescentes, independentemente do sexo, os praticantes de karatê apresentaram maior massa óssea em relação ao grupo controle, sendo o BTT mais sensível para essa avaliação.

## INTRODUCTION

The maximum bone mass reached by the young adult (peak bone mass) is strongly influenced by the process of sexual maturation, as normal growth and interaction between endogenous (hereditary and endocrine features) and exogenous (nutrition and physical activity) factors take place.^1^ ^,^ ^2^ In childhood and adolescence, bone mass increases gradually, reaching 90% of its peak, with predominance of bone formation over bone absorption,^3^ so it is a turning point for bone response to physical exercise.^4^

Studies have shown that athletes have more bone mass compared to non-athletes, especially those who practice high-impact sports, as the occurrence of microfractures in bone tissue stimulate osteogenesis.^5^ ^,^ ^6^ Bone mass gain seems to depend on the sport one plays.^6^ ^,^ ^7^ ^,^ ^8^ ^,^ ^9^ ^,^ ^10^ ^,^ ^11^ However, specific physical exercises (type, intensity, frequency and duration) required to improve it in childhood and adolescence have not been outlined yet.^10^

Karate^6^, a high-impact sport, is the most popular martial art in the world, practiced by children, adolescents, adults, and the elderly.^12^ It involves basic techniques such as kicks, punches, and blocks (offensive and defensive) divided into two styles: Kata (imaginary fight) and Kumite (combat).^13^ The modality engages several muscle groups with complex movements and fast accelerations and decelerations.^14^ The short-duration attack and defense techniques are characterized by execution in maximum intensity with short intervals, which makes it comparable to an intermittent and intense exercise.^14^

Studies that have evaluated bone mass in karate practitioners-either by quantitative ultrasound (QUS) of phalanges^15^ or dual energy X-ray absorptiometry (DXA)-point out the benefits of this modality for bone health.^16^ ^,^ ^17^ However, they are scarce. Only one of them is known to have included males within a very large age range (from 7 to 61 years) and used QUS of phalanges by the Amplitude-Dependent Speed of Sound (AD-SoS in m/s) parameter.^14^ Two studies assessed bone mass in children and adolescents who would practice martial arts (not exclusively karate) using DXA.^15^ ^,^ ^16^ Thus, the purpose of this study was to evaluate bone mass of children and adolescents practicing karate by QUS of phalanges based on AD-SoS and Bone Transmission Time (BTT, in µs).

## METHOD

This is a cross-sectional case-control study with children and adolescents aging 6 to 16 years of both genders. The case group (karate practitioners) was composed of students enrolled in Karate in 2014 at selected gym centers of all seven cities of western Paraná (Cascavel, Capanema, Matelândia, Medianeira, São Miguel do Iguaçu, Palotina, and Toledo), totaling 258 (98 females and 160 males), of which 162 (63%) (52 females and 110 males) participated in the study. The control group was assembled from a database of our laboratory with healthy students enrolled in municipal schools of Cascavel (Paraná), all being evaluated by the same method^17^ and paired in the proportion of two controls for each case, according to gender, age, weight, height, and body mass index (BMI), with total of 326 participants (110 females and 216 males). Inclusion criteria for selection of karate practitioners was: being properly enrolled in the karate program at gym centers of the western region of Paraná, aging between 6 and 16 years, being healthy, not using continuous medication, and having the informed consent form signed by their parents or guardians (absence of signature was the reason 37% of karate practitioners did not participate in the study). Control group selection included the following criteria: attending school in the city of Cascavel (western Paraná), aging between 6 and 16 years, presenting complete data in the research group database, being healthy, not using continuous medication, and having the informed consent form signed by their parents or guardians.

The informed consent was granted by the gym center management, parents and/or caregivers of karate practitioners, school principals, parents and/or caregivers of subjects in control groups. This research was approved by the Research Ethics Committees of Universidade Federal de Santa Catarina (control group: 131/2006) and Faculdade Assis Gurgacz (karate practitioners: 191/2013, control group: 220/2008). Data were collected at the gym center or at the schools.

The weight (kg) was evaluated with a digital scale of the brand Tanita^®^ graduated with 100 g; height (cm), was measured with a Seca^®^ wall-mounted stadiometer graduated with 1 mm. From these data, BMI (kg/m^2^) was calculated. The BMI values were transformed in Z score and sorted according to the International Obesity Task Force (IOTF):^18^ normal+overweight (female <2.19; male <2.29) and obese (female ≥2.19; male ≥2.29). This BMI category grouping (normal+overweight group and obese group) was based on the physiological point of view that overweight does not exert positive influence on one’s bone mass, approaching normal threshold, but obesity does impact bone mass both positively (weight) and negatively (inflammatory processes).^19^

Bone parameters were assessed by QUS of the phalanges (DBM Sonic Bone Profiler BP-01 IGEA^®^, Capri, Italy). Evaluations complied with the manufacturer’s standard protocol, being performed on the proximal phalange distal metaphysis of the non-dominant hand, from the second to the fifth finger. Parameters evaluated were AD-SoS (m/s) and BTT (µs). AD-SoS is the measure of range between the first signal transmitted and the last one received, influencing soft tissue; BTT reflects bone properties regardless of effects on soft tissue, thus being considered more accurate for this evaluation. The absolute values of AD-SoS and BTT measures were converted to Z scores, as in the reference study by Barkmann *et al*.^20^ Based on these results, all subjects were sorted into two groups: below expectation (Z scores ≤-2.0) or adequate (Z scores >-1.99). Upon QUS evaluation, intra- and inter-observer variation coefficients in our research group were 0.6 and 1.5%, respectively.^17^

The Shapiro-Wilk test was used to verify data normality. As they did not present a normal distribution, the variables were shown as median, minimum and maximum values. Mann-Whitney test was used to compare groups according to gender, while the chi-square or Fischer’s exact tests were used to compare the frequencies of individuals with adequate bone mass and BMI. The Spearman’s test was applied to assess correlation between bone parameters, age, and anthropometric measures (weight, height, BMI, BMI Z-score). After data transformation, stepwise multiple linear regression was applied to evaluate the effects of each independent variable on bone parameters. P-value ≤0.05 was adopted as significance level. Afterwards, sample effect was studies based on the power of each test and using the Standard Power Calculation method and the R test, with level of significance set at α = 0.05 for all tests. SPSS program version 20.0 was used to process statistics.

## RESULTS

Table 1 shows overall characteristics of karate practitioners and control groups according to gender. Female karate practitioners had higher BMI and BTT Z scores when compared to controls; Control group, on its turn, had superior AD-SoS and AD-SoS Z scores. Male karate practitioners presented BTT and BTT Z score higher than the values presented by the control group. All other variables were similar in both groups in relation to gender.

**Table 1:**
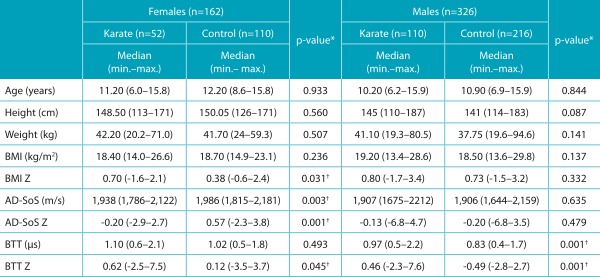
Overall features of the sample comprising karate practitioners and control subjects according to gender.

AD-SoS: Amplitude-Dependent Speed of Sound; BTT: Bone Transmission Time; BMI: body mass index; *Mann-Whitney test; ^†^Sample power >99%.

Table 2 shows the frequency of young karate practitioners and control subjects sorted by gender and according to their bone mass and BMI classifications. Only male karate subjects had normal bone mass more commonly, as shown by BTT Z score when compared to same-gender subjects in control group (Table 2).

**Table 2:**
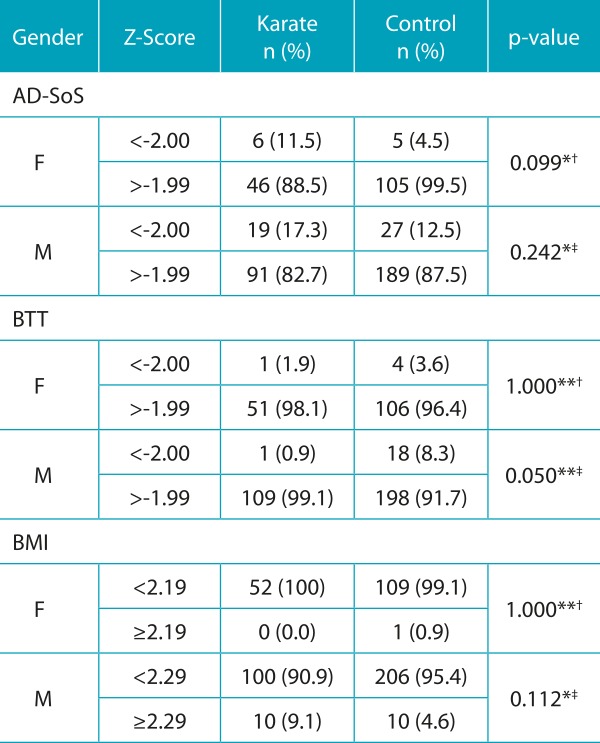
Relative and absolute frequencies with regard to Z-score, Amplitude-Dependent Speed of Sound, Bone Time Transmission and body mass index values according to gender, karate practice, and control group.

AD-SoS: Amplitude-Dependent Speed of Sound; BTT: Bone Transmission Time; BMI: body mass index; *chi-square; **Fischer’s test; ^†^Sample power at 26%; ^‡^Sample power at 46%.

When it comes to correlations between bone and anthropometric parameters, none was found between BMI and AD-SoS of males practicing karate. All other variables had moderate to high/significant correlations, usually above 0.40. Exceptions were BMI and AD-SoS in females practicing karate, weight and AD-SoS in males practicing karate; BMI and BTT in males practicing karate, BMI and AD-SoS and MTB in male subjects in control group; although positive, such correlations were low (Table 3).

**Table 3:**
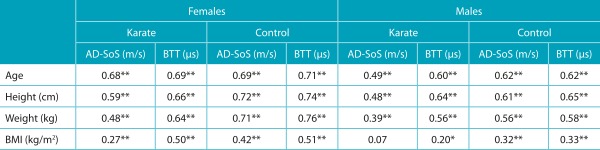
Correlations of Amplitude-Dependent Speed of Sound and Bone Transmission Time with age, height, weight, and body mass index according do gender, karate practice, and control group.

Spermann’s test: *p<0.05; **p<0.01; BTT: Bone Transmission Time; AD-SoS: Amplitude-Dependent Speed of Sound; BMI: body mass index.

Table 4 shows results of the multiple linear regression model for AD-SoS and BTT variables of both genders. With the exception of females who practiced karate, age was the variable that best explained AD-SoS variation, with explanatory power between 26 and 42%. Similar results were obtained in BTT for both groups, and best-prediction variables were weight for females (r^2^ = 0.45 for karate group, r^2^ = 0.51 for control groups) and height for males (r^2^ = 0.36 for karate group, r^2^ = 0.42 for control group).

**Table 4:**
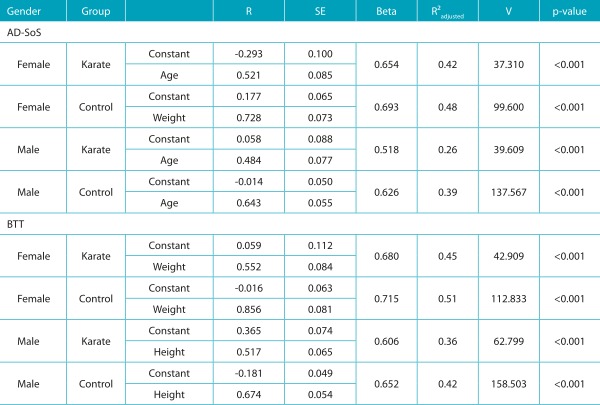
Results of the multiple linear regression model for Amplitude-Dependent Speed of Sound and Bone Transmission Time variables among females (F) and males (M) by group, according to anthropometric variables.

BTT: Bone Transmission Time; AD-SoS: Amplitude-Dependent Speed of Sound; R: linear regression coefficient; SE: standard error; V: analysis of variance (ANOVA).

## DISCUSSION

In our study, young karate practitioners of both genders had more bone mass, adjusted for age and gender (BTT Z-score) and compared to the control group. Females’ weight and males’ height were the best prediction anthropometric data.

In the literature, only a study by Drozdzowska *et al*.^14^ evaluated bone mass using QUS in karate practitioners. However, it only included males aged 7 to 61 years, and the bone mass was evaluated by AD-SoS only. Results showed that the duration, the frequency, and period of beginning of activity were determining for bone mass gain, and that is why adults were shown to benefit more from karate practice. In our study, there was also an increase in bone mass with daily practice of exercises and techniques related to karate among the elderly, but such benefit was also seen in children and adolescents of both genders.

Although the results of our study indicate higher AD-SoS among karate practitioners as opposed to control group, one must note that BTT is more sensitive for bone mass evaluation when assessed by QUS in relation to AD-SoS and, for this reason, this parameter was included in the study. Such variables show the ultrasound velocity in the bone, but AD-SoS is the interval measured between the first signal transmitted and the last one received with influence on soft tissues. BTT, on its turn, shows bone properties regardless of the confounding effect of soft tissue.^20^ The difference between AD-SoS and BTT may explain the higher values of ADS-SoS (absolute value and Z-score) found in females of the control group as opposed to karate practitioners, once subjects in karate group presented higher BMI scores-which indicates the influence of soft tissue on the evaluation. However, the BTT Z-score was higher in the karate group compared to control group for both genders.

Karate engages several muscle groups with complex movements and rapid acceleration and deceleration movements by displacement techniques and different postures.^14^ The short-duration attack and defense techniques are characterized by execution in maximum intensity with short intervals, which makes the modality comparable to an intermittent and intense exercise.^14^ Allied to maximum intensity, these techniques reinforce the findings of the present study, according to which bone mass gain and stress take place through constant executions. In 2008, Koropanovski *et al*. established that upper limb techniques are often predominant (89.1%) compared to lower limb techniques (8.4%).^21^ Punching techniques are more efficient, with greater chance of reaching the target, compared kicking techniques. This may explain the wider use of the upper limbs during karate.^22^ These findings are similar to ours: the predominant use of upper limbs in karate and techniques engaging them lead to a higher bone mass index.

Recently, Nasri *et al*.^16^ assessed the effects of combat sports (judo, karate, kyokushinkai karate, boxing, and kung fu) on bone mineral density as measured by DXA in adolescents^16^ ^,^ ^17^ and reported greater bone mass in athletes compared to group control. In 2001, Andreolli *et al*.^23^ evaluated bone mineral density in male young adults (judo, karate, water polo practitioners and non-practitioners) and described greater bone mineral density by DXA in karate and judo practitioners compared to practitioners of aquatic polo and non-practitioners of such sports, drawing attention to the difference in bone health pointed out regardless of evaluation method and age group. QUS rather than DXA technique was used in our study, and we also stated bone mass gain in karate practitioners compared to control group, which means that the method is suitable for bone tissue assessment with the benefit of easy applicability, handling and portability.

In 2011, Tenforde and Fredericson^6^ made a review of articles on bone mineral density assessed by DXA in athletes aged 10 to 30 years and reported a higher density related to the practice of high-impact sports (gymnastics, hurdles, judo, karate, volleyball and other involving jumping), as well as sports with frequent but not constant impact (soccer, basketball, racquet sports, aerobics, and speed skating) compared to non-impact sports (swimming, water polo, and cycling). Although QUS and DXA techniques that evaluate different bone mass data, there is a certain correlation between their results that has already been demonstrated in different studies and confirmed by Baroncelli *et al*.^20^ Therefore, the findings of the present study, in which QUS of phalanges was used, more specifically to evaluate BTT, may be comparable to the abovementioned studies on bone mineral density and DXA in karate practitioners.^6^ ^,^ ^16^ ^,^ ^17^ ^,^ ^24^

Löfgren *et al*.^4^ conducted an intervention study with children of both genders aging 7 to 9 years. They reported that children submitted to the intervention program presented higher bone mass index upon DXA, without risk of fractures, compared to the control group, confirming that doing exercises, such as karate or other type of physical activity of impact, is fundamental for the health of children and adolescents.

In Brazil, studies using QUS of phalange in healthy children and adolescents of both genders showed the influence of age, weight and height on the increase of bone mass.^1^ ^,^ ^25^ ^,^ ^26^ ^,^ ^27^ Such investigations showed that increase in bone mass was influenced by age, puberty, and height^1^, and that AD-SoS and Ultrasound Bone Profile Index (UBPI) were higher according to age and puberty.^25^ Increase in AD-SoS was dependent on lean and fat masses^26^ and the AD-SoS and UBPI were shown to be higher according to age, puberty and height^27^. In 2014, Krahenbühl *et al*.^28^ made a systematic review of articles relating to bone mass assessed by QUS in children and adolescents and found that both AD-SOS and BTT increased with age, just like anthropometric measures of weight and height; these findings are similar to those reported in studies that have been conducted with different ethnicities. In this sense, the relation of variables with bone parameters is explained by normal growth for pediatric age, and they can vary from person to person. As noted in our study, age and height have influence on this.

Consequently, increase in bone mass in children and adolescents related to age and puberty is expected and has been well documented by Lappe *et al*.^2^ The authors evaluated bone mineral density of 1,743 children and adolescents of both genders aged between 6 and 16 years and described the effects of weight-lifting exercises on bone mass during puberty. Also important to note is that puberty is comprised of physical changes, such as weight gain and increase in height, influenced by the hormonal stimuli of each gender, predominating androgenic stimulation in males, with gain of lean mass and height, and estrogenic in females, with gain of weight and fat mass.^28^ This explains the findings of or study, that is, the link between bone mass and anthropometric features, especially the BTT Z-score related to weight in girls and height in boys.

However, some of the limitations of our study are worth mentioning, non-evaluation of puberty stages and of duration and intensity of karate practice included. It is, though, a pioneering study on bone mass assessed by QUS, with a significant number of children and adolescents practicing karate in relation to a control group.

Bone mass evaluation-in karate practitioners initially when performing basic techniques and, later on, during classes and progress of bands-can help maintain the health of the practitioner and, therefore, to improve their quality of life, confirming the importance and benefits of combat sports in pediatric age. In this study, BTT was shown to be the most adequate and useful parameter to conduct evaluations with karate practitioners due to its greater sensitivity for bone tissue assessment. Considering the lack of evidence on this matter in the scientific literature, this study may be used as example for further research on combat sports (high impact), to elucidate their benefits for bone health. Conclusion is that karate practitioners in this group of children and adolescents, regardless of gender, were shown to present higher bone mass index compared to the control group.

## References

[1] Santos KD, Petroski EL, Ribeiro RR, Guerra G (2009). Bone quantity and quality in Brazilian female schoolchildren and adolescents. J Bone Miner Metab.

[2] Lappe JM, Watson P, Gilsanz V, Hangartner T, Kalkwarf HJ, Oberfield S (2015). The longitudinal effects of physical activity and dietary calcium on bone mass accrual across stages of pubertal development. J Bone Miner Res.

[3] Mora S, Gilsanz V (2003). Establishment of peak bone mass. Endocrinol Metab Clin North Am.

[4] Löfgren B, Dencker M, Nilsson JÅ, Karlsson MK (2012). A 4 year exercise program in children increases bone mass without increasing fracture risk. Pediatrics.

[5] Greene DA, Naughton GA (2006). Adaptive skeletal responses to mechanical loading during adolescence. Sports Med.

[6] Tenforde AS, Fredericson M (2011). Influence of sports participation on bone health in the young athlete: a review of the literature. PM R.

[7] Gruodyte R, Jürimäe J, Cicchella A, Stefanelli C, Passariello C, Jürimäe T (2010). Adipocytokines and bone mineral density in adolescent female athletes. Acta Paediatr.

[8] Ito IH, Mantovani AM, Agostinete RR, Costa P, Zanuto EF, Christofaro DG (2016). Practice of martial arts and bone mineral density in adolescents of both sexes. Rev Paul Pediatr.

[9] Kohrt WM, Bloomfield SA, Little KD, Nelson ME, Yingling VR (2004). American College of Sports Medicine Position Stand: physical activity and bone health. Med Sci Sports Exerc.

[10] Gracia Marco L, Moreno LA, Ortega FB, León F, Sioen I, Kafatos A (2011). Levels of physical activity that predict optimal bone mass in adolescents: the HELENA study. Am J Prev Med.

[11] Koropanovski N, Berjan B, Bozic PR, Pazin N, Sanader A, Jovanovic S (2011). Anthropometric and physical performance profiles of elite karate kumite and kata competitors. J Hum Kinet.

[12] Imamura H, Yoshimura Y, Uchida K, Nishimura S, Nakazawa AT (1998). Maximal oxygen uptake, body composition and strength of highly competitive and novice karate practitioners. Appl Human Sci.

[13] Milanez VF, Dantas JL, Christofaro DG, Fernandes RA (2012). [Heart rate response during a karate training session]. Rev Bras Med Esporte.

[14] Drozdzowska B, Münzer U, Adamczyk P, Pluskiewicz W (2011). Skeletal status assessed by quantitative ultrasound at the hand phalanges in karate training males. Ultrasound Med Biol.

[15] Nasri R, Hassen Zrour S, Rebai H, Najjar MF, Neffeti F, Bergaoui N (2013). Grip strength is a predictor of bone mineral density among adolescent combat sport athletes. J Clin Densitom.

[16] Nasri R, Zrour SH, Rebai H, Neffeti F, Najjar MF, Bergaoui N (2015). Combat sports practice favors bone mineral density among adolescent male athletes. J Clin Densitom.

[17] Gonçalves EM, Ribeiro RR, Carvalho WR, Moraes AM, Roman EP, Santos KD (2015). Brazilian pediatric reference data for quantitative ultrasound of phalanges according to gender, age, height and weight. PLoS One.

[18] Cole TJ, Lobstein T (2012). Extended international (IOTF) body mass index cut offs for thinness, overweight and obesity. Pediatr Obes.

[19] Barkmann R, Rohrschneider W, Vierling M, Tröger J de TF, Cadossi R (2002). German pediatric reference data for quantitative transverse transmission ultrasound of finger phalanges. Osteoporos Int.

[20] Baroncelli GI (2008). Quantitative ultrasound methods to assess bone mineral status in children: technical characteristics, performance, and clinical application. Pediatr Res.

[21] Koropanovski N, Dopsaj M, Jovanovic S (2008). Characteristics of pointing actions of top male competitors in karate at world and European level. Braz J Biomotricity.

[22] Chaabène H, Franchini E, Miarka B, Selmi MA, Mkaouer B, Chamari K (2014). Time motion analysis and physiological responses to karate official combat sessions: is there a difference between winners and defeated karatekas?. Int J Sports Physiol Perform.

[23] Andreoli A, Monteleone M, Van Loan M, Promenzio L, Tarantino U, Lorenzo A (2001). Effects of different sports on bone density and muscle mass in highly trained athletes. Med Sci Sports Exerc.

[24] Ribeiro RR, Guerra G, Azevedo Barros A (2009). Bone mass in schoolchildren in Brazil: the effect of racial miscegenation, pubertal stage, and socioeconomic differences. J Bone Miner Metab.

[25] Carvalho WR, Gonçalves EM, Ríbeiro RR, Farias ES, Carvalho SS, Guerra G (2011). Influence of body composition on bone mass in children and adolescents. Rev Assoc Med Bras.

[26] Moraes AM, Gonçalves EM, Barbeta VJ, Guerra G (2013). Cross sectional study of the association of body composition and physical fitness with bone status in children and adolescents from 11 to 16 years old. BMC Pediatr.

[27] Krahenbühl T, Gonçalves EM, Costa ET, Barros A (2014). Factors that influence bone mass of healthy children and adolescents measured by quantitative ultrasound at the hand phalanges: a systematic review. Rev Paul Pediatr.

[28] Loomba Albrecht LA, Styne DM (2009). Effect of puberty on body composition. Curr Opin Endocrinol Diabetes Obes.

